# Active colonization dynamics and diversity patterns are influenced by dendritic network connectivity and species interactions

**DOI:** 10.1002/ece3.1020

**Published:** 2014-03-12

**Authors:** Mathew Seymour, Florian Altermatt

**Affiliations:** 1 Department of Aquatic Ecology, Eawag: Swiss Federal Institute of Aquatic Science and TechnologyÜberlandstrasse 133, 8600, Dübendorf, Switzerland; 2 Department of Environmental Systems Science, ETH ZentrumCHN H41, 8092, Zürich, Switzerland

**Keywords:** Connectivity, dispersal, landscape structure, linear network, microcosm, protist, river-like

## Abstract

Habitat network connectivity influences colonization dynamics, species invasions, and biodiversity patterns. Recent theoretical work suggests dendritic networks, such as those found in rivers, alter expectations regarding colonization and dispersal dynamics compared with other network types. As many native and non-native species are spreading along river networks, this may have important ecological implications. However, experimental studies testing the effects of network structure on colonization and diversity patterns are scarce. Up to now, experimental studies have only considered networks where sites are connected with small corridors, or dispersal was experimentally controlled, which eliminates possible effects of species interactions on colonization dynamics. Here, we tested the effect of network connectivity and species interactions on colonization dynamics using continuous linear and dendritic (i.e., river-like) networks, which allow for active dispersal. We used a set of six protist species and one rotifer species in linear and dendritic microcosm networks. At the start of the experiment, we introduced species, either singularly or as a community within the networks. Species subsequently actively colonized the networks. We periodically measured densities of species throughout the networks over 2 weeks to track community dynamics, colonization, and diversity patterns. We found that colonization of dendritic networks was faster compared with colonization of linear networks, which resulted in higher local mean species richness in dendritic networks. Initially, community similarity was also greater in dendritic networks compared with linear networks, but this effect vanished over time. The presence of species interactions increased community evenness over time, compared with extrapolations from single-species setups. Our experimental findings confirm previous theoretical work and show that network connectivity, species-specific dispersal ability, and species interactions greatly influence the dispersal and colonization of dendritic networks. We argue that these factors need to be considered in empirical studies, where effects of network connectivity on colonization patterns have been largely underestimated.

## Introduction

Understanding colonization and invasion dynamics is a long-standing question in ecology (Fisher 1937; Skellam 1951; Elton 1958). On the one hand, colonization of empty habitat is the most important process that counteracts the negative effects of extinctions on biodiversity (Hanski 1999; Clobert et al. 2012). On the other hand, the spread of invasive species has negative effects on native species, and there are strong incentives to understand, and eventually slow down, invasion fronts (Hastings et al. 2005). It is generally accepted that colonization dynamics are influenced by several abiotic and biotic factors and are related to the spatial (network) structure of the environment, but also depend on interspecific interactions and stochastic effects (Melbourne and Hastings 2009; Clobert et al. 2012). However, most empirical and theoretical studies on dispersal dynamics have focused on dispersal in highly simplified habitat systems, such as two-dimensional networks (i.e., lattice-like) (Okubo et al. 1989; Hanski 1999) or randomized networks (Melbourne and Hastings 2009). It was found that network connectivity, defined as the extent to which habitat sites within a network are connected to one another and the ease (speed) with which species move between the sites (Newman 2010), and subsequent changes therein alter colonization patterns and ecological dynamics (Holland and Hastings 2008).

Such studies are meaningful in explaining dispersal and colonization dynamics of organisms in two-dimensional ecosystems such as forests or grasslands. For example, Okubo et al. (1989) used simplified networks to predict the spatial spread of gray squirrels in Britain and theoretically related the spread to both spatial landscape structure as well as interspecific interactions. However, the findings may not be applicable to more complex network hierarchies found in nature, such as river networks, mountain ranges, or cave networks, which have dendritic connectivity (Rodriguez-Iturbe and Rinaldo 1997). Dendritic river networks are of specific interest in ecology and conservation biology as they harbor a disproportionally high species richness compared with other natural systems and have been greatly altered by human activity (Vinson and Hawkins 1998; Grant et al. 2007; Clarke et al. 2008; Vörösmarty et al. 2010; Altermatt 2013; Altermatt et al. 2013; Göthe et al. 2013; Peterson et al. 2013). In such dendritic networks, network connectivity may have a greater, and possibly different, impact on colonization and community dynamics compared with lattice-like or linear networks (Fagan 2002; Campos et al. 2006; Bertuzzo et al. 2007; Grant et al. 2007; Rodriguez-Iturbe et al. 2009). Furthermore, river systems are subject to strong habitat modifications and serve as dispersal pathways for pathogens and invasive species (Bertuzzo et al. 2010; Lynch et al. 2011; Mari et al. 2011), making a causal understanding of how network structure affects colonization and invasion dynamics of great importance.

Recent theoretical studies postulate that network connectivity has direct effects on species persistence and diversity (Fagan 2002; Labonne et al. 2008; Muneepeerakul et al. 2008; Brown and Swan 2010). Specifically, Fagan (2002) and Labonne et al. (2008) showed theoretically that the increased connectivity of dendritic networks can increase the persistence of metapopulations, assuming low dispersal. These studies compared persistence dynamics of one species in linear networks versus symmetrically bifurcating dendritic networks. Cuddington and Yodzis (2002) expanded to species interactions and found that the coexistence of predator and prey is prolonged in dendritic networks compared with linear networks. Theoretical work on diversity and multispecies coexistence in dendritic networks has recently gained increased attention (e.g., Muneepeerakul et al. 2008; Rodriguez-Iturbe et al. 2009; Bertuzzo et al. 2010), but largely lacked experimental verifications. Recently, Carrara et al. (2012, 2014) gave the first experimental demonstration on how dendritic network structure results in characteristic diversity patterns. They found that increased network connectivity (here lattice versus dendritic networks) increased average species diversity per site and decreased species diversity between sites. They also found species richness is greater at highly connected sites (i.e., “main river stem”) compared with less connected sites (i.e., “headwaters”) within dendritic networks. However, Carrara et al. (2012, 2014) used experimentally controlled dispersal rates between distinct sites and treated all species equally. This approach removes the influence of species-specific dispersal ability, which is a crucial aspect of these species’ biology (Cadotte and Fukami 2005; Limberger and Wickham 2011), and also making it an important factor in our understanding of diversity in dendritic networks. Studies using active colonization in dendritic networks are thus needed to improve our understanding of colonization dynamics in complex hierarchical networks, such as river networks (Rodriguez-Iturbe and Rinaldo 1997; Grant et al. 2007).

Comparative case examples are consistent with the above-mentioned theoretical expectations, also strongly indicating that the component of active dispersal is crucial. For example, Leuven et al. (2009) observed a 10-fold increase in non-native species actively colonizing the river Rhine drainage systems since 1825. The increase was found to be highly correlated with increased network connectivity between the Rhine and other previously distant, or isolated, drainage systems due to the construction of river canals and transportation of species along shipping routes. This highlights that network connectivity of rivers not only follows strict hierarchical patterns that directly influence dispersal pathways (Rodriguez-Iturbe and Rinaldo 1997; Fagan 2002; Grant et al. 2007; Muneepeerakul et al. 2008), but that riverine communities may be especially sensitive to changes in the network connectivity.

Additionally, not only network connectivity, but also species interactions may affect colonization dynamics. In the absence of spatial structure, species competing for the same resources are expected to competitively exclude each other, such that weaker competitors go extinct, thereby reducing the local species diversity (Hardin 1960). However, the inclusion of spatial factors is expected to prolong the persistence of weaker competitors within a metacommunity through processes such as the competition–colonization trade-offs, thereby maintaining greater species diversity and promoting diversity among neighboring sites (Hastings 1980). In a classic study, Okubo et al. (1989) showed how species interactions slow down the spread of species into previously occupied space using theoretical models and comparing them with empirical data. However, the effect of species interactions on colonization dynamics may be more complex and not univocal. For example, French and Travis (2001) experimentally showed that species interactions (parasitoid/prey dynamics) can increase, decrease, or have no effect on spread rates and that spread rates are highly species dependent, using a two-patch system. Again, no experimental studies have yet tested the effect of dendritic network structure and species interactions on colonization dynamics.

The main objective of our study was to assess the influence of how linear versus dendritic network connectivity affects species colonization dynamics and subsequent diversity patterns, with and without the presence of species interactions. We used experimental microcosm networks and a set of protist and rotifer species. This model system has been shown to be good for testing ecological concepts derived from natural systems (Warren 1996; McGrady-Steed et al. 1997; Cadotte 2007; Haddad et al. 2008; Altermatt et al. 2011b; Altermatt and Holyoak 2012; Carrara et al. 2012). The study species not only cover a wide range of known trait values, but their interactions are also well documented (Cadotte et al. 2006; Altermatt et al. 2011a). We specifically wanted to further previous work by implementing different realistic network connectivities, which allow active colonization, and asked the following questions: (1) Does the increased connectivity of dendritic networks, relative to linear networks, also increase the species-specific colonization rates at the network scale and what are the subsequent effects on diversity? (2) How are intraspecific colonization dynamics altered when a single species or a community of species colonizes linear versus dendritic networks? (3) Can we predict active colonization patterns with independently measured species traits? Based on these questions and previous work, we hypothesized: (1) colonization rates, measured as the number of sites occupied per day, proceeds faster, and species diversity will be greater in dendritic networks, compared with linear networks, due to increased connectivity in the dendritic network (Lynch et al. 2011). Specifically, the higher persistence of species in dendritic versus linear networks is predicted to be a direct consequence of network structure (Fagan 2002); (2) the presence of species interactions to decrease individual species colonization rates and decrease community diversity due to competitive interactions, compared with when absent (Okubo et al. 1989; Cuddington and Yodzis 2002); (3) species traits, such as growth rate, cell size, and carrying capacity, which are traits associated with dispersal ability (Cadotte 2006), to positively affect colonization dynamics.

## Material and Methods

**Figure 1:**
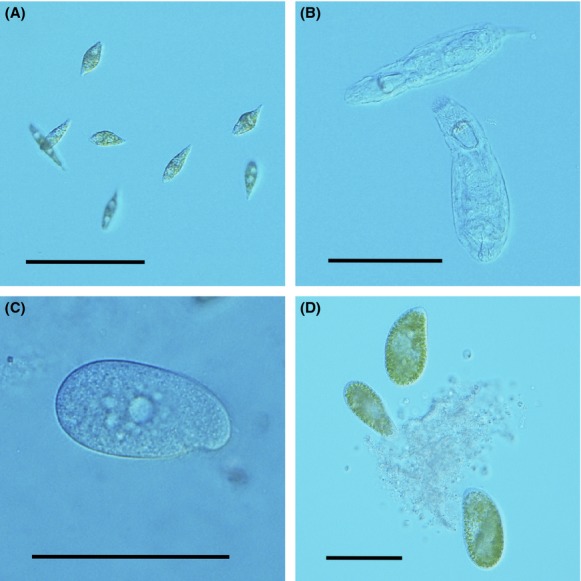
Four of the seven protist and rotifer species used in the microcosm experiments. The species are *Euglena gracilis* (A), *Cephalodella* sp. (B, the rotifer), *Colpidium* sp. (C), and *Paramecium bursaria* (D). The scale bar corresponds to 100 *μ*m.

For our experiments, we used a set of six protist species (*Chilomonas* sp., *Colpidium* sp.*, Euglena gracilis*, *Euplotes aediculatus*, *Paramecium bursaria*, and *Tetrahymena* sp.) and one rotifer species (*Cephalodella* sp.), which colonized aquatic microcosms (Fig. 1). Species were cultured in protist medium, along with a set of common freshwater bacteria (*Serratia fonticola*, *Brevibacillus brevis*, and *Bacillus subtilis*) as a food source. Protist medium was made by adding 0.2 g/L protozoan pellet, supplied by Carolina Biological Supply, to tap water, autoclaving, and then cooling to room temperature before use. *Chilomonas* sp. and *Tetrahymena* sp. were supplied by Carolina Biological Supply, whereas all other species were originally isolated from a natural pond (McGrady-Steed et al. 1997) and have been used for other studies (Haddad et al. 2008; Altermatt et al. 2011b; Carrara et al. 2012). All protist and the rotifer species are primarily bacterivores; however, some species (e.g., *Eup. aediculatus*) may also prey on smaller protist species, such as *Chilomonas* sp. Furthermore, *Eug. gracilis*, *Eup. aediculatus*, and *P. bursaria* are capable of photosynthesis.

### Experimental setup

**Figure 2:**
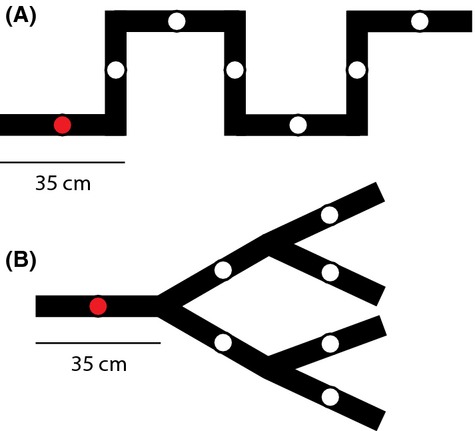
Schematic illustration of the experimental networks used in this study, made of silicon tubing. (A) Linear network and (B) dendritic network. Both continuous network types were of equal total volume (125 mL) and equal length of tubing. Each connecting section (i.e., edge) was 35 cm long and had a single opening (indicated with a circle) in the middle of the section for sampling. Tubing width and openings are given to scale. Red circles indicate the starting site where protists and the rotifer species were introduced at the onset of the experiments.

We used two different types of networks, a linear channel (linear network, Fig. 2A) and a bifurcating dendritic network (Fig. 2B). Both network types were made of silicon tubing connected with T- and Y-connectors. The two network types had the same total length (245 cm) and total volume (125 mL). Seven T-shaped openings were evenly distributed across both network types to allow sampling, and the ends of the networks were secured to prevent leakage using metal clamps. Networks were assembled and autoclaved 1 day prior to experimental use. The environmental conditions throughout the experiment were the same for all microcosm units using a climate chamber (20°C and constant fluorescent lighting). The algebraic connectivity, which accounts for the centrality and eccentricity of a given network, was calculated using the second lowest eigenvalue of the Laplace matrix of the respective network (Chung 1997).

### Single-species colonization

**Figure 3:**
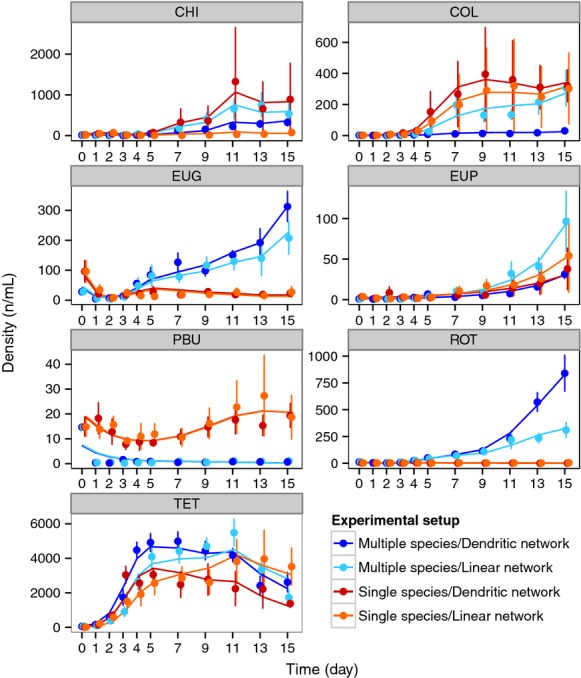
Density (individuals per mL) at the network level over time. Each panel depicts the results for a different species, with the species’ name indicated in the header of each panel (CHI =* Chilomonas* sp., COL = *Colpidium* sp., EUG = *Euglena gracilis*, EUP = *Euplotes aediculatus*, PBU = *Paramecium bursaria*, ROT = *Cephalodella* sp., and TET = *Tetrahymena* sp.). Points indicate the density (*y*-axis) at each time point sampled (*x*-axis). The colors correspond to the different experimental setups (orange = single-species in linear landscape, red = single-species in dendritic landscape, light blue = multiple-species in linear landscape, and dark blue = multiple-species in dendritic landscape). The upper and lower whiskers correspond to the 1.5 times interquartile range. The lines are GAM model fits of individual species’ models (Table S1), fitted to each of the separate treatment combinations.

We set up an experiment to measure how individual species independently spread and colonize the two network types. Due to the large number of samples and processing time required, we used a block design (experimental units conducted at different time points) for the single-species colonization experiment. For each block, we had 14 networks (seven dendritic and seven linear). In total, we used four blocks. In each block, all species were included, such that each species spread in one dendritic and one linear network. One week prior to each block setup, we established fresh cultures for each of the protist and rotifer species. We added 25 mL of each species’ stock culture to 125 mL of fresh protist medium, in previously autoclaved Erlenmeyer flasks containing two wheat seeds, and allowed the species to grow to carrying capacity. The densities of the protist and rotifer species were checked for each inoculation to ensure subsequent densities were comparable and related to the species’ carrying capacity (Fig. 3). This method was preferred over using the same number of individuals for all species, as it reflects a more realistic interspecies comparison based on differences in growth rate and carrying capacities. Twelve hours prior to species inoculation, networks were filled with 125 mL of protist medium and inoculated with the above described set of freshwater bacteria.

To measure colonization over time, we individually released the protist and rotifer species at the starting site of each network (Fig. 2). For each network, a clamp was placed 2 cm to the right of the starting site to prohibit initial protist movement beyond the first site during the setup procedure. We removed 1.5 mL of medium from the starting site and replaced it with 1.5 mL of a given species’ culture, which had grown to carrying capacity. Right thereafter, silicon stoppers were used to close all openings to prevent laminar flow, and the clamp removed to allow for active colonization. Silicon stoppers were then removed to allow air exchange with the medium throughout the experiment. All sites were routinely sampled by removing 0.5 mL medium from each network site, which was replaced with 0.5 mL of fresh, autoclaved, protist medium inoculated with bacteria. Sampling occurred every 24 h for 5 days and then every 48 h for an additional 10 days. To avoid laminar flow during sampling, we used silicon stoppers, which we placed on all site openings except the one being sampled. Once the medium was replaced, we added a stopper to the sampled site and removed the stopper from the next site we needed to sample from, and so on. We used a Nikon SMZ1500 stereomicroscope (Nikon Corporation, Tokyo, Japan) to measure density of protists and rotifers (individuals per mL). If species density was too high to be accurately counted, we diluted the sample until an appropriate measure could be taken (Altermatt et al. 2011b).

### Multiple-species colonization

In contrast to the single-species colonization setup (absence of species interactions), in the multiple-species colonization setup, all species were released together in each network, such that they could interact during the subsequent spread (presences of species interactions). The initial setup of the multiple-species colonization setup was identical to the single-species colonization setup, with five replicates of each network type used. In order to inoculate each network with the same number of individuals per species at the start of the experiment, we concentrated individuals in the community by mixing 1.5 mL of each species together and removing as much of the medium as possible, without affecting the species’ total abundances, using a syringe and 20-*μ*m filters. We then clamped the section of the tubing 2 cm to the right of the first network site, removed medium, and replaced it with the multiple-species community inoculum. Species abundances were checked prior to inoculation to ensure they were comparable to densities in the single-species experiment (Fig. 3). All sampling and counting methods were conducted in the same way as in the single-species experiment.

### Statistical analysis

All statistical analyses were performed using the program R, version 2.15.1 (R Development Core Team 2012).

Species abundances, as measured with the method previously mentioned, were used to assess temporal intraspecific colonization patterns and interspecific interactions occurring between the single-species (virtual) and multiple-species (observed) setups. We assessed the relationship between the species’ abundance (response variable) and the three explanatory variables time, presence/absence of species interactions (single- vs. multiple-species setups), and network type (linear vs. dendritic), by fitting generalized additive models (GAM) with a Poisson distribution, using a backward model fitting approach for each species using the mgcv package in the program R (Wood 2011). We included time as a smoothing term given the nonlinear relationship of species abundance over time.

To describe the temporal change in interspecific species abundances across the networks (i.e., species diversity over time and space), we calculated the mean local species richness, Jaccard similarity (Faith et al. 1987), and Pielou's evenness.(Jost 2010), using the package vegan in R (Oksanen et al. 2009). Whereas species richness is a standard method to assess local (site) species diversity and measured as the number species at a given site(Whittaker 1956), Jaccard similarity is an established method to assess among site species diversity and is calculated using 1 – [2B/(1 + B)] where B is the Bray–Curtis dissimilarity (Faith et al. 1987; Condit et al. 2002). Jaccard similarity is a metric measurement and is often preferred over Bray–Curtis similarity, which is semimetric. Pielou's evenness is used as a measure of distribution of relative species abundances in a community (Jost 2010). Pielou's evenness is calculated using J = H/log (S), where H is the Shannon–Weaver diversity index and S is the number of species. All three diversity measures were calculated for the multiple-species setup by calculating the mean diversity measure for each network at each time point and then taking the mean of the network-type replicates (i.e., dendritic and linear network replicates were averaged separately). The measurements from the multiple-species replicates represent our observed communities, whereby species interactions were present. For the single-species setup, we performed the same calculation as the multiple-species setup, but pooled the species-specific replicates within each block together to calculate a virtual community (i.e., absence of species interactions) value for linear and dendritic networks, and calculated the final mean across the blocks. This gave us a real (observed) community value (multiple-species setup) and an expected (virtual) community value (single-species setup) for our diversity measures in the linear and dendritic networks and allowed us to compare colonization dynamics with and without species interactions. In the multiple-species setup, species interactions could take place as all species were present, while in the single-species setup, these interactions were absent. By calculating a virtual community value, we could assign the effect of species interactions on community composition and colonization dynamics. We then assessed relationship of species richness, Jaccard similarity, and Pielou's evenness relative to time, species community setup (presence/absence of species interactions) and network type using a GAM model with a backward model fitting approach. We included time as a smoothing term due to the nonlinear relationship of the response variable over time (Zuur et al. 2009).

Protist and rotifer species traits were previously measured in other experimental studies (Cadotte and Fukami 2005; Haddad et al. 2008; Altermatt et al. 2011a; Carrara et al. 2012; Giometto et al. 2013). We used these previously measured species traits, including growth rate, cell mass, and carrying capacity, and tested for correlations with occupancy. We did this comparison at the time when variation between networks was strongest (day nine), using linear regression.

Finally, to characterize colonization patterns in the single- and multiple-species community setups, we calculated the rate of spread for each species in each experimental setup based on how much time was required to reach maximum occupancy (number of sites). Sites were considered occupied when a species was observed present at a given site when sampled, irrespective of the density. We performed a two-way ANOVA, by fitting a linear model using the lm function in R, to determine whether there was an effect of species or network type on the rate of spread in the networks.

## Results

Species abundances (mean density at network level) showed strong differences between species and generally increased over time (Fig. 3, Table S1). Species abundances were significantly different between experimental setups (single- versus multiple-species communities, *P* < 0.001). Most species also showed significant differences (*P* < 0.001) in abundances between network types (linear versus dendritic) over time (Fig. 3, Table S1).

**Figure 4:**
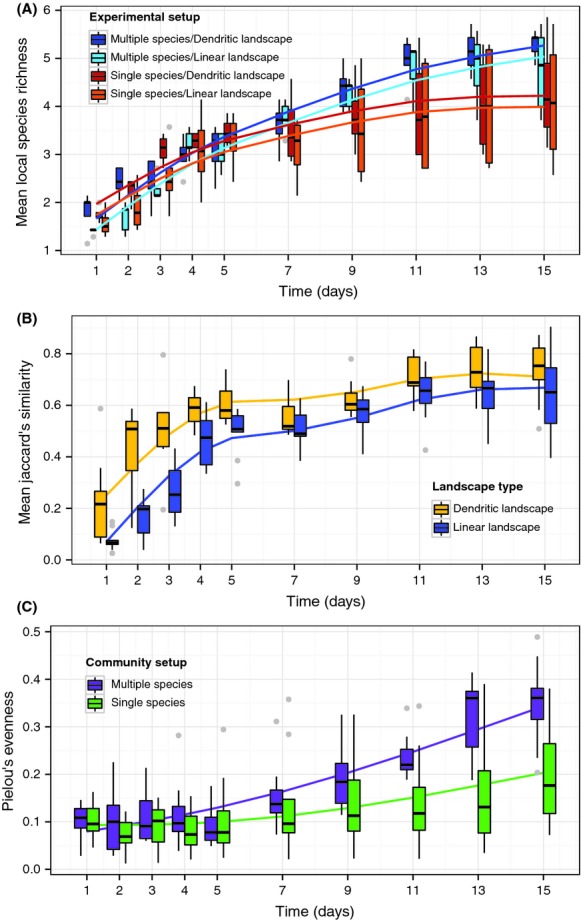
(A) Mean local species richness (*α*-diversity) over time for each network type used in the single- and multiple-species community setups (orange = single-species in linear network, red = single-species in dendritic network, light blue = multiple-species in linear network and dark blue = multiple-species in dendritic network). *α*-diversity of the single-species treatment was calculated by virtually pooling the species from individual experimental blocks and averaging across all blocks (i.e., it is a “virtual” community value). The lines are GAM model fits, fitted to each of the separate treatment combinations. The upper and lower whiskers correspond to the 1.5 times interquartile range. (B) Mean Jaccard similarity index over time for each network type used in the single- and multiple-species community setups (linear networks = blue, dendritic networks = yellow). Pairwise Jaccard similarity was calculated for all community pairs within a network, and the mean value thereof is used here. Jaccard similarities of the single-species community setups were calculated by pooling the species from individual experimental blocks and averaging across all blocks (i.e., it is a “virtual” community value). The lines are GAM model fits, fitted to each of the separate treatment combinations that were significant in the final model. (C) Mean Pielou's evenness over time for each network setup in linear and dendritic networks (single-species setup = green, multiple-species setup = purple). The lines are GAM model fits, fitted to each of the separate treatment combinations that were significant in the final model.

Similarly, diversity patterns showed strong differences over time and experimental setups (Fig. 4 and Table 1). Mean local species richness increased over time for all experimental settings (Fig. 4A). There were significant effects of network type and community setup on mean local richness (Table 1). In multiple-species communities, mean local richness increased faster compared with the calculated values from the single-species communities (i.e., the “virtual” community; Table 1, Fig. 4A). We found a significantly greater increase in species richness over time in the dendritic networks compared with the linear networks. There was also a significant time by community setup interaction, meaning that mean local species richness increased over time at a different rate for the single-and multiple-species community setups.

**Table 1:** Generalized additive models (GAM), explaining local species richness (A), Jaccard similarity (B), and Pielou's evenness (C) over time. Model estimates and significances of the smoothing terms are given for the final most parsimonious models. *R*^2^ values of the final models are 0.73 for species richness, 0.74 for Jaccard similarity, and 0.50 for Pielou's evenness.

(A) Species richness	Estimate	SE	*t*-value	*P*-value
(Intercept)	0.51	0.13	3.97	<0.001
Time	0.44	0.02	22.59	<0.001
Community setup	0.4	0.17	2.31	0.022
Network type	−0.23	0.09	−2.45	<0.001
Time × community setup	−0.1	0.02	−4.66	<0.001
Approximate significance of smooth terms:				
	edf	Ref.df	*F*	*P*-value
s(Time)	3.191	4.005	30.03	<0.001
(B) Jaccard similarity	Estimate	SE	*t*-value	*P*-value
(Intercept)	0.25	0.03	7.77	<0.001
Time	0.02	0	4.93	<0.001
Community setup	0.04	0.02	2.36	0.019
Network type	0.19	0.03	6.48	<0.001
Time × network type	−0.01	0	−2.81	0.005
Approximate significance of smooth terms:				
	edf	Ref.df	*F*	*P*-value
s(Time)	4.62	5.636	41.63	<0.001
(C) Pielou's evenness	Estimate	SE	*t*-value	*P*-value
(Intercept)	0.02	0.01	2.22	<0.001
Time	0.02	0	16.96	<0.001
Community setup	0.02	0.02	1.21	0.228
Time × community setup	−0.01	0	−4.51	<0.001
Approximate significance of smooth terms:				
	edf	Ref.df	*F*	*P*-value
s(Time)	1.8	2.32	5.56	0.003

Jaccard similarity increased over time for all experimental settings (Fig. 4B). The main finding was that Jaccard similarity increased significantly faster in the dendritic networks compared with the linear networks, at the beginning of the experiment, but both became similar toward the end of the experiment (Fig. 4B, Table 1). The statistical model supports this observation, such that time (*P* < 0.001), network type (*P* < 0.001), and the interaction time x network type (*P* = 0.005) were significant in the model (Table 1). This means that Jaccard similarity differed between sampling times and network types and that the relationship of similarity over time differed between the linear and dendritic networks. There was no significant effect of community setup (single- vs. multiple-species) on Jaccard similarity.

Evenness increased over time for all experimental settings with the multiple-species community setup showing higher evenness toward the end of the experiment compared with the single-species experiment (Fig. 4C). The statistical model showed that time (*P* < 0.001) and the interaction time x community setup (*P* < 0.001) were significant to the model (Table 1). These results indicate that evenness not only differed between sampling times and community setups, but that evenness became more different between the multiple- and single- species setups over time. Network type, however, did not significantly affect evenness (Table 1).

Species differed in their rate of spread (i.e., number of sites occupied per day) and generally spread faster in the dendritic networks compared with the linear networks (Fig. S1). We found significant effects of species identity (*P* < 0.001 both in the single-species setup and in the multiple-species setup), network type (*P* = 0.035 in the single-species setup and *P* < 0.001 in the multiple-species setup), and species × network type interaction (*P* = 0.021 in the single-species setup and *P* < 0.001 in the multiple-species setup) on the rate of spread (Table S2).

**Figure 5:**
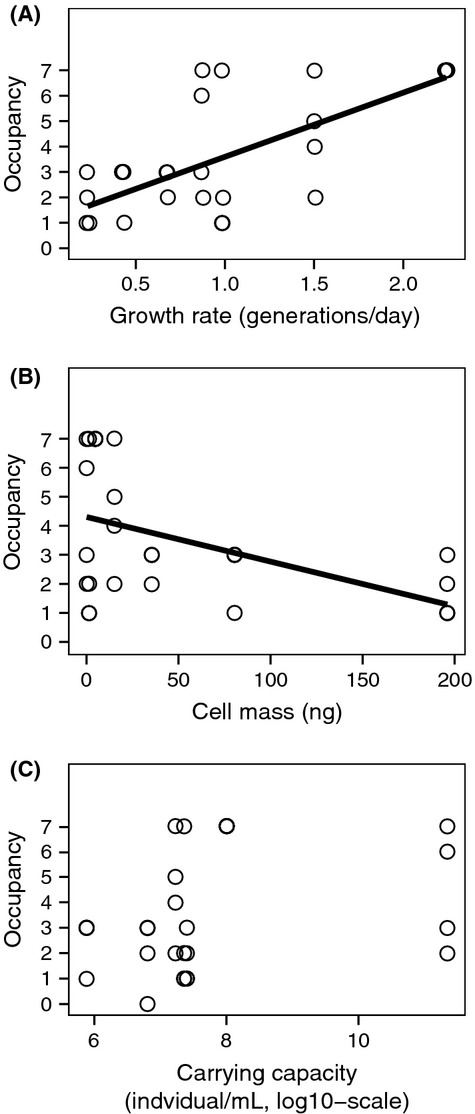
Occupancy (number of sites occupied) at day nine versus species-specific trait values (A) growth rate, (B) cell mass, and (C) carrying capacity. Data are from the single-species setups in the linear landscape. For significant correlations, we added the fitted curve from the corresponding linear model.

We found a significant positive correlation between a species’ growth rate and occupancy (*F*_1,26_ = 23.8, *P* < 0.001, Fig. 5A), a significant negative correlation between cell mass and occupancy (*F*_1,26_ = 6.16, *P* = 0.02, Fig. 5B), and no significant correlation between carrying capacity and occupancy at day nine of the experiment (*F*_1,26_ = 0.85, *P* = 0.37, Fig. 5C).

## Discussion

We experimentally showed that network connectivity, specifically linear versus dendritic networks (Fig. 2), influenced abundance, diversity, and colonization dynamics of actively spreading species (Figs. 2, 3, and S1). The different species cover a large range of trait values (Fig. 5), including growth rate or cell mass (Altermatt et al. 2011a), which makes our experimental findings not only consistent with theoretical expectations, but also robust in showing the influence of dendritic network structure on species colonization dynamics. Fagan (2002) theoretically showed that metapopulation persistence of single species can be either enhanced or reduced in dendritic versus linear networks, depending on the details of dispersal (i.e., dispersal rate and direction). With our experiment, covering a wide range of species trait values, we experimentally demonstrate enhanced persistence and diversity in dendritic networks as a direct consequence of increased connectivity of these networks compared with linear networks of identical size (but with inherently larger maximum distance).

As expected, mean local species richness increased throughout the experiment (Fig. 4A), as a consequence of species colonizing the network from one site of origin. However, species richness was consistently higher in the dendritic versus the linear networks (Fig. 4A). Thus, for networks of the same total size (i.e., number of sites), the dendritic structure substantially increased diversity compared with the linear structure. A possible explanation, also consistent with theoretical expectation, is that increasing network connectivity increases (re)colonization rates and persistence of species (Hess, 1996, Fagan 2002). While a similar effect has been found for dendritic systems with externally manipulated dispersal (Carrara et al. 2012, 2014), we show that it is also present when species can move actively and unconstrained. The inclusion of active, unconstrained movement is important, as dispersal and subsequent occupancy patterns are not independent of species traits (Fig. 5). This dependence is disrupted in experiments that apply passive, externally constrained dispersal. We also found an increase in mean community similarity (Jaccard similarity index as a measure of *β*-diversity) over time, with a stronger effect expressed in dendritic networks (Fig. 4B). Again, the higher Jaccard similarity in the dendritic network compared with the linear network is likely due to the increased connectivity in the dendritic (algebraic network connectivity = 0.27) compared with the linear networks (algebraic network connectivity = 0.20; see also Cuddington and Yodzis 2002; Fagan 2002; Lynch et al. 2011). Specifically, Lynch et al. (2011) showed that increasing network connectivity increased species’ dispersal potential, resulting in increased community similarity across a river network.

Furthermore, species abundances and diversity differed between single- and multiple-species setups over time (Figs. 3, 4). Species interactions are a major factor in understanding dispersal and have been shown to greatly influence dispersal and metacommunity dynamics (Leibold et al. 2004). We found that the presence of species interactions promoted dispersal rates and subsequently affected colonization dynamics. This is surprising and not directly expected (French and Travis 2001; Cadotte 2007). For example, Okubo et al. (1989) predicted slower spread and lower diversity due to species interactions, while we found greater species richness and evenness in the multiple-species setups compared with the virtual single-species setup (Figs. 3, 4). A possible explanation is that increased dispersal (and subsequent colonization) in the presence of other species is not directly compensated by higher competitive exclusion over the same timescale (i.e., that effects on dispersal versus effects on competitive exclusion happen at different timescales), eventually resulting in higher diversity. An important implication thereof is that a temporally aligned spread of multiple (potentially invasive and interacting) species in river networks may result in a higher overall invasion success, a pattern that had indeed been observed in some river systems (e.g., Leuven et al. 2009).

The species used in our experiment, which primarily feed on bacteria, have been observed to competitively exclude each other in previous single-patch experiments (Cadotte 2007; Haddad et al. 2008). However, no species went extinct within the networks in the multiple-species setups during the course of the experiment (i.e., γ-diversity = 7 species throughout the experiment). We found some species densities were influenced by the presence or absence of species interaction (Fig. 3), but not all species coexisted locally. This finding is consistent with theoretical predictions that the connectivity of dendritic networks (compared with linear networks) may have a positive influence on the stability of communities within metapopulations (Fagan 2002) or metacommunities, as used here. Specifically, the network connectivity may allow inferior competitive or prey species to persist due to spatial dynamics and competition–colonization trade-offs (Holyoak and Lawler 1996). Based on previous species competition rankings in Haddad et al. (2008), we found species which were deemed highly competitive, including *Euplotes* and *Cephalodella* sp.*,* were also slow colonizers (Figs. 5 & S2),whereas species that had low competitive rankings in previous studies, including *Chilomonas*, *Euglena, Tetrahymena* and *Colpidium,* were much faster colonizers. These finding are consistent with competition–colonization trade-offs reported in previous active colonization studies (e.g., Cadotte 2007; Limberger and Wickham 2011) using simplified landscape, but unobservable in studies using complex network structure with passive dispersal (e.g., Carrara et al. 2012, 2014).

## Conclusion

While recent theoretical dispersal studies have incorporated complex network connectivites, empirical data on dispersal in dendritic networks are still lagging behind (but see Mari et al. 2011; Barták et al. 2013). This is problematic, as natural riverine networks are highly diverse (e.g., Vörösmarty et al. 2010), and changes in connectivity directly influence biodiversity (e.g., Leuven et al. 2009; Grant et al. 2012). Experimental microcosm studies offer an opportunity to bridge theoretical models (e.g., Fagan et al. 2002; Muneepeerakul et al. 2008; Grant et al. 2012) with empirical field studies (e.g., Leuven et al. 2009), allowing a better understanding of dispersal in dendritic networks.

We experimentally demonstrated that higher connectivity (dendritic compared with linear) promoted species colonization, with subsequent effects on metacommunity diversity patterns. Most importantly, dendritic networks exhibited higher diversity over time to otherwise identical linear networks (Fig. 4), and that this effect was even stronger when species interactions were present. We also found that specific dispersal-related traits correlate with colonization rate and species diversity across the networks.

Overall, our findings indicate network connectivity, and species interactions greatly influence the active dispersal and colonization of species in dendritic networks. We argue these factors need to be considered in empirical studies, where effects of network connectivity and species interactions on dispersal and species diversity patterns have long been underestimated.

## Supporting Information

**Table S1.** Generalized additive models (GAM), explaining individual species abundances (density) relative to experimental setup (single- vs. multiple-species communities), time, and network type (linear vs. dendritic networks). **Table S2.** Spread rates for single- and multiple-species experimental setups. **Figure S1.** Rate of spread in the linear and dendritic network types in the single- (left) and multiple-species (right) experimental setups. **Figure S2.** Proportion of network occupied over time for single- and multiple-species experimental setups (left and right column, respectively).
