# Supplementary material for: Active colonization dynamics and diversity patterns are influenced by dendritic network connectivity and species interactions
Source: Ecol Evol. 2014 Mar 12;4(8):1243–54. doi: 10.1002/ece3.1020 (PMC4020686; doi:10.1002/ece3.1020)
Supplement: Supplementary file 1 — Table S1. Generalized additive models (GAM), explaining individual species abundances (density) relative to experimental setup (single- vs. multiple-species communities), time, and network type (linear vs. dendritic networks). Table S2. Spread rates for single- and multiple-species experimental setups. Figure S1. Rate of spread in the linear and dendritic network types in the single- (left) and multiple-species (right) experimental setups. Figure S2. Proportion of network occupied over time for single- and multiple-species experimental setups (left and right column, respectively). [file ece30004-1243-sd1.docx]

*Electronic Appendix A: Supplementary Information file*

**Active colonization dynamics and diversity patterns are**

**influenced by dendritic network connectivity and species interactions**

Mathew Seymour^1,2,*^, Florian Altermatt^1^

1. Department of Aquatic Ecology, Eawag: Swiss Federal Institute of Aquatic Science and Technology, Überlandstrasse 133, 8600 Dübendorf, Switzerland,

2. Department of Environmental Systems Science, ETH Zentrum, CHN H41, 8092 Zürich, Switzerland

* corresponding author: Mat.Seymour@eawag.ch

**Supplementary information to illustrate species-differences among the different experimental setup conditions.**

Supplementary Table S1. Generalized additive models (gam), explaining individual species abundances (density) relative to experimental setup (single- vs. multiple-species communities), time, and network type (linear vs. dendritic networks). Model estimates of the most parsimonious model, and significances of the smoothing terms are given for each species. Species names are given in the header (A–G) of the respective model (CHI = *Chilomonas* sp., COL= *Colpidium* sp., EUG = *Euglena gracilis*, EUP = *Euplotes aediculatus*, PBU = *Paramecium bursaria*, ROT = *Cephalodella* sp., and TET = *Tetrahymena* sp*.*)

|  | Estimate | Std. Error | zvalue | Pr(>\|z\|) |
| --- | --- | --- | --- | --- |
| A) CHI |  | | | |
| Setup | 2.109137 | 0.108698 | 19.404 | <0.001 |
| Time | 0.543196 | 0.009083 | 59.801 | <0.001 |
| Network Type | 1.770371 | 0.110343 | 16.044 | <0.001 |
| Setup x Time | -0.08482 | 0.008796 | -9.644 | <0.001 |
| Setup x Network Type | -3.28825 | 0.06853 | -47.983 | <0.001 |
| Time x Network Type | -0.08542 | 0.008935 | -9.559 | <0.001 |
|  |  |  |  |  |
| Approximate significance of the smoothing term: | | | |  |
|  | edf | Ref.df | Chi.sq | p-value |
| s(Time) | 7.962 | 7.999 | 2301 | <0.001 |
| Deviance explained | 94% |  |  |  |
|  |  |  |  |  |
| B) COL |  | | | |
| Setup | 3.412551 | 0.158459 | 21.536 | <0.001 |
| Time | 0.164334 | 0.019352 | 8.492 | <0.001 |
| Network Type | 1.90013 | 0.146745 | 12.949 | <0.001 |
| Setup x Time | -0.05368 | 0.011427 | -4.698 | <0.001 |
| Setup x Network Type | -2.45297 | 0.106888 | -22.949 | <0.001 |
| Time x Network Type | 0.032322 | 0.009764 | 3.31 | <0.001 |
|  |  |  |  |  |
| Approximate significance of the smoothing term: | | | |  |
|  | edf | Ref.df | Chi.sq | p-value |
| s(Time) | 7.266 | 7.697 | 980.3 | <0.001 |
| Deviance explained | 98% |  |  |  |
|  |  |  |  |  |
| C) EUG |  | | | |
| Setup | 0.635261 | 0.090726 | 7.002 | <0.001 |
| Time | 0.636976 | 0.006412 | 99.343 | <0.001 |
| Network Type | 0.031434 | 0.082042 | 0.383 | <0.001 |
| Setup x Time | -0.23474 | 0.011346 | -20.689 | <0.001 |
| Setup x Network Type |  |  |  |  |
| Time x Network Type | -0.02324 | 0.007952 | -2.922 | <0.001 |
|  |  |  |  |  |
| Approximate significance of the smoothing term: | | | |  |
|  | edf | Ref.df | Chi.sq | p-value |
| s(Time) | 7.867 | 7.992 | 2392 | <0.001 |
| Deviance explained | 94% |  |  |  |
|  |  |  |  |  |
| D) EUP |  | | | |
| Setup | 1.15237 | 0.30836 | 3.737 | <0.001 |
| Time | 0.19083 | 0.02705 | 7.054 | <0.001 |
| Network Type | 0.1642 | 0.31513 | 0.521 | <0.001 |
| Setup x Time | -0.07513 | 0.02361 | -3.183 | <0.001 |
| Setup x Network Type | -0.57413 | 0.18965 | -3.027 | <0.001 |
| Time x Network Type | 0.06402 | 0.0235 | 2.724 | <0.001 |
|  |  |  |  |  |
| Approximate significance of the smoothing term: | | | |  |
|  | edf | Ref.df | Chi.sq | p-value |
| s(Time) | 1.698 | 2.11 | 3.24 | 0.212 |
| Deviance explained | 95% |  |  |  |
|  |  |  |  |  |
| E) PBU |  | | | |
| Setup | 0.21634 | 4.436 | 9.16E-06 | <0.001 |
| Time | 0.03551 | 1.069 | 0.285 | <0.001 |
| Setup x Time | 0.0468 | 4.807 | 1.53E-06 | <0.001 |
|  |  |  |  |  |
| Approximate significance of the smoothing term: | | | |  |
|  | edf | Ref.df | Chi.sq | p-value |
| s(Time) | 3.692 | 4.538 | 108.7 | <0.001 |
| Deviance explained | 87% |  |  |  |
|  |  |  |  |  |
| F) ROT |  | | | |
| (Intercept) |  |  |  |  |
| Setup | -1.22144 | 0.32987 | -3.703 | <0.001 |
| Time | 0.59284 | 0.01066 | 55.592 | <0.001 |
| Network Type | 0.57254 | 0.14353 | 3.989 | <0.001 |
| Setup x Time | -0.31625 | 0.03132 | -10.096 | <0.001 |
| Setup x Network Type | 0.57541 | 0.29073 | 1.979 | 0.048 |
| Time x Network Type | -0.09883 | 0.01148 | -8.61 | <0.001 |
|  |  |  |  |  |
| Approximate significance of the smoothing term: | | | |  |
|  | edf | Ref.df | Chi.sq | p-value |
| s(Time) | 7.78 | 7.972 | 512.2 | <0.001 |
| Deviance explained | 99% |  |  |  |
|  |  |  |  |  |
| G) TET |  | | | |
| Setup | -0.45209 | 0.015563 | -29.049 | <0.001 |
| Time | 1.168518 | 0.00151 | 773.885 | <0.001 |
| Network Type | -0.72403 | 0.015918 | -45.486 | <0.001 |
| Setup x Time | 0.007316 | 0.001648 | 4.439 | <0.001 |
| Setup x Network Type | 0.221669 | 0.01276 | 17.372 | <0.001 |
| Time x Network Type | 0.076763 | 0.001649 | 46.56 | <0.001 |
|  |  |  |  |  |
| Approximate significance of the smoothing term: | | | |  |
|  | edf | Ref.df | Chi.sq | p-value |
| s(Time) | 7.892 | 7.993 | 499118 | 0.212 |
| Deviance explained | 93% |  |  |  |

Supplementary Table S2. Spread rates for single- and multiple-species experimental setups. Summary statistics are given for each factor in the two-way Anova, both for the single-species and the multiple-species setups. Included are the degrees of freedom (DF), F-statistic (F-value) and p-value for each factor in the model.

|  | DF | F-value | p-value |
| --- | --- | --- | --- |
| A) Single-species setup |  | | |
| Species | 6 | 8.22 | <0.001 |
| Network Type | 1 | 4.73 | 0.035 |
| Species x Network Type | 6 | 2.83 | 0.021 |
| Residuals | 42 |  |  |
| B) Multiple-species setup |  | | |
| Species | 6 | 9.37 | <0.001 |
| Network Type | 1 | 25.50 | <0.001 |
| Species x Network Type | 6 | 5.91 | <0.001 |
| Residuals | 56 |  |  |


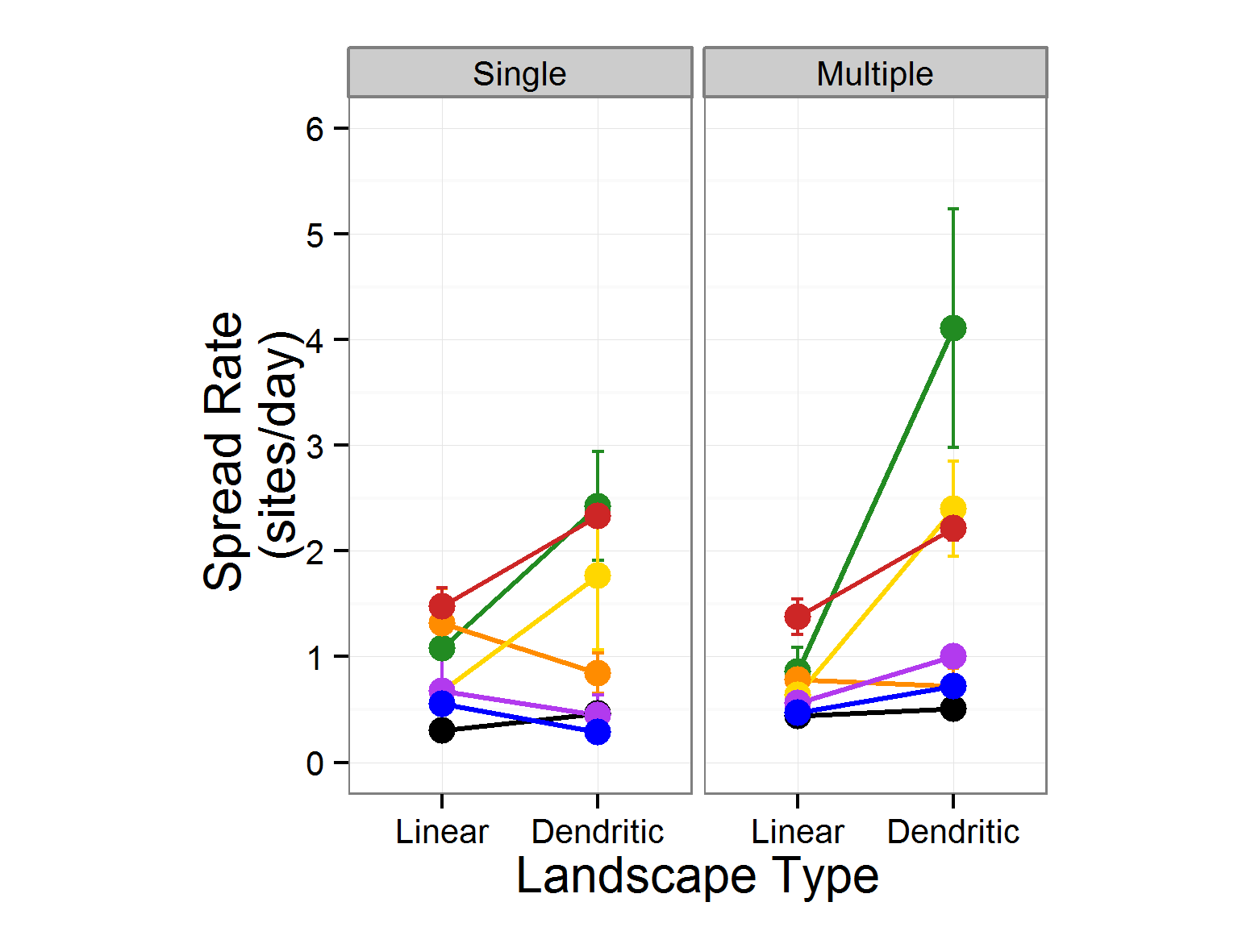


Supplementary Figure S1. Rate of spread in the linear and dendritic network types in the single- (left) and multiple-species (right) experimental setups. Each color represents a different species. The upper and lower whiskers correspond to the 1.5 times interquartile range.


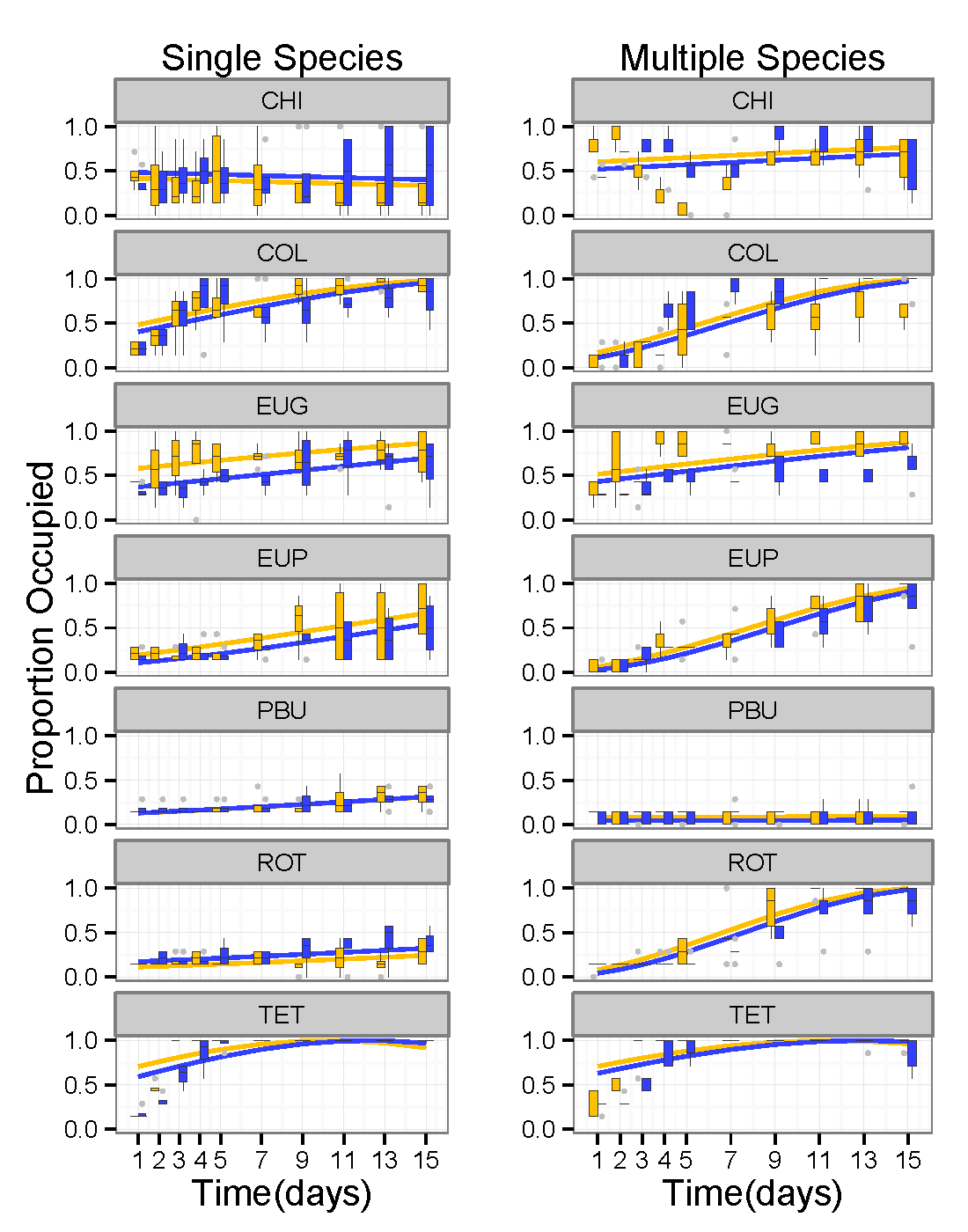


Supplementary Figure S2. Proportion of network occupied over time for single- and multiple-species experimental setups (left and right column respectively). Each panel depicts the results for a different species, with the species name indicated in the header of each panel (CHI = *Chilomonas* sp*.*, COL= *Colpidium sp.,* EUG = *Euglena gracilis*, EUP = *Euplotes aediculatus*, PBU = *Paramecium bursaria*, ROT = *Cephalodella* sp. (a rotifer), and TET = *Tetrahymena* sp*.*). Upper and lower sections of the boxplots correspond to the first and third quartiles. Whiskers correspond to the points that are within 1.5 times the interquartile range. The lines are model fits, with proportion occupied as the response variable and time as the explanatory variable, for the linear (yellow) and dendritic (blue) network type.
